# Stochastic master equation for early protein aggregation in the transthyretin amyloid disease

**DOI:** 10.1038/s41598-020-69319-x

**Published:** 2020-07-24

**Authors:** Ruo-Nan Liu, Yan-Mei Kang

**Affiliations:** School of Mathematics and Statistics, Xi’an Jiaotong University, Xi’an, 710049 Shaanxi China

**Keywords:** Nonlinear dynamics, Stochastic modelling

## Abstract

It is significant to understand the earliest molecular events occurring in the nucleation of the amyloid aggregation cascade for the prevention of amyloid related diseases such as transthyretin amyloid disease. We develop chemical master equation for the aggregation of monomers into oligomers using reaction rate law in chemical kinetics. For this stochastic model, lognormal moment closure method is applied to track the evolution of relevant statistical moments and its high accuracy is confirmed by the results obtained from Gillespie’s stochastic simulation algorithm. Our results show that the formation of oligomers is highly dependent on the number of monomers. Furthermore, the misfolding rate also has an important impact on the process of oligomers formation. The quantitative investigation should be helpful for shedding more light on the mechanism of amyloid fibril nucleation.

## Introduction

The aggregation of soluble proteins or protein fragments into non-soluble fibrillary polymers is a hallmark of a range of increasingly prevalent and devastating human disorders such as Alzheimer’s disease, Parkinson’s disease, Huntington’s disease and familial amyloid polyneuropathy^1–4^. In these amyloidoses, dozens of proteins or protein components of disease-associated amyloid deposits have been identified so far, for instance, amyloid-beta peptide, alpha-synuclein, Huntingtin and transthyretin. Among these components, transthyretin, as a homotetrameric protein which is mainly synthesized in the liver, the choroid plexus and the retina, is implicated in several amyloid pathologies including familial amyloid polyneuropathy, familial amyloid cardiomyopathy, senile systemic amyloidosis and central nervous system selective amyloidosis^4–6^.

Increasing studies suggest oligomers, the aggregation intermediate species, are correlated with the cellular toxicity in various forms of amyloidogenesis^7–9^, which motivates the researchers to disclose how the oligomeric species are formed during the early stages of amyloid aggregation. In most of the known mechanisms for amyloid aggregation^10–19^, the aggregation process is initiated by a coarse-grained “primary nucleation” reaction step, which proceeds via oligomeric intermediates^10–19^. Primary nucleation as a critical step in the amyloid formation cascade refers to the initial formation of nuclei through self-organization is characterized by the presence of a free energy barrier^20,21^. During this early stage, there are still no amyloid fibrils.

Mathematical model researches for amyloid aggregation^15,22–24^ are helpful in shedding light on experimental observations and developing therapeutic strategies. Knowles et al*.*^15^ developed an analytical solution to the kinetic of the complex self-assembly of filamentous molecular structures. Meisl et al*.*^22^ described a framework to elucidate a molecular mechanism of protein aggregation by ways of quantitative kinetic assays and global fitting. Michaels et al*.*^24^ presented an experimental and theoretical approach to drive the dynamics of oligomers during the aggregation of Alzheimer's Aβ42 peptide. It is worth emphasizing that most kinetic models of protein oligomers in the literature are deterministic.

Due to the intrinsic stochasticity in biochemical reaction^25–27^, however, the deterministic model cannot always accurately capture the essential dynamics of amyloid aggregation^28,29^. Note that oligomers are the most toxic structures and play potential role as a target in drug discovery^30^, so we take the early amyloid aggregation process of making oligomers as the main research focus, with transthyretin oligomers formed from the aggregation of at most six monomers^31,32^. To capture the stochastic effects in early amyloid aggregation, we build a mathematical model of chemical master equation, which is well accepted as probabilistic description in well-mixed and dilute condition, to describe the aggregation of monomers into oligomers. And then, we use lognormal closure moment method rather than direct simulation to acquire the time dependent evolution of low-order statistical moments, and we emphasize that the semi-analytic moment method can greatly reduce the computational cost^33–36^.

The paper is organized as following. In “Chemical master equation model” we describe the stochastic method of modeling the aggregation of monomers into oligomers and present moment closure method for computing the time evolution of stochastic models. In “Results”, we apply the moment closure method to the stochastic model and examine the numerical results by comparing with the simulated results. Moreover, we investigate the impact of the number of monomers and the misfolding rate on the aggregation dynamics. The conclusions are drawn in last section.

## Chemical master equation model

### Build model of oligomers formation

Amyloid formation is considered to be a complex protein aggregation process, which consists of a range of molecular processes. In the process of protein filaments formation, primary nucleation is usually followed by growth^10–19^ and self-replication through secondary pathways in some cases^11,17,18^. The nucleation phase corresponds to the period where monomers undergo conformational changes and self-associate to form the oligomeric nuclei. This phase is determined by the critical concentration of nuclei and generally considered to be thermodynamically unfavorable^21^. The growth/elongation phase represents the period in which the oligomeric nuclei acting as seeds rapidly grow and form mature fibrils. Considering the nucleation phase is an essential part of the overall aggregation process, as a large variety of oligomeric species are gradually formed^21^, so we ignore elongation but focus on the process of nucleation below.

**Figure 1:**
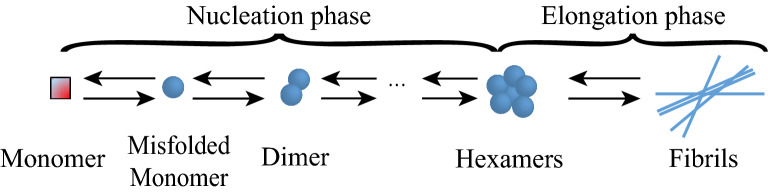
The two phases (nucleation phase and growth/elongation phase) of amyloid formation.

Following Refs.^32,37^, we suppose that (1) the monomers undergo conformational changes into misfolded monomers which then are polymerized into small polymers involving diameter (two monomers), trimer (three monomers), etc.; (2) the small polymers polymerize (depolymerize) into bigger (smaller) structures by attaching (losing) one misfolded monomer; (3) the maximum oligomer size is limited to six since this is relevant for transthyretin oligomers^31,32^. Then, the process of making oligomers can be illustrated in Fig. 1. Let $M_{{0}} ,M_{1} ,\;M_{2} , \ldots ,M_{{6}}$ denote monomers, misfolded monomers, dimers, triamers, tetramers, pentamers, hexamers, respectively. The mathematical scenario is shown in Table 1.

**Table 1:** Kinetics of monomers aggregation.

Reaction	Propensity function	Reaction	Propensity function
$M_{{0}} \to M_{1}$	$K_{0} M_{{0}}$	$M_{1} \to M_{{0}}$	$K_{{6}} M_{1}$
$M_{1} + M_{1} \to M_{2}$	$K_{1} M_{1} M_{1}$	$M_{2} \to M_{1} + M_{1}$	$K_{{7}} M_{{2}}$
$M_{1} + M_{2} \to M_{3}$	$K_{2} M_{2} M_{1}$	$M_{3} \to M_{1} + M_{2}$	$K_{{8}} M_{{3}}$
$M_{1} + M_{3} \to M_{4}$	$K_{3} M_{3} M_{1}$	$M_{4} \to M_{1} + M_{3}$	$K_{{9}} M_{{4}}$
$M_{1} + M_{4} \to M_{5}$	$K_{4} M_{4} M_{1}$	$M_{5} \to M_{1} + M_{4}$	$K_{{{10}}} M_{{5}}$
$M_{1} + M_{{5}} \to M_{{6}}$	$K_{5} M_{5} M_{1}$	$M_{{6}} \to M_{1} + M_{{5}}$	$K_{{{11}}} M_{{6}}$

First, we briefly present the stochastic formulation inspired by Ref.^35^. If a model has *L* biochemical reactions and *N* molecular species, it can be written as

1 $$s_{l1} X_{1} + \cdots + s_{lN} X_{N} \to r_{l1} X_{1} + \cdots + r_{lN} X_{N} \,,\;1 \le l \le L$$

where $s_{li}$ and $r_{li}$ are the coefficients of change of the molecular species $X_{i}$ involved in the *l*th reaction. We define the stoichiometric matrix as $S =( {S_{lj} } )$ where $S_{lj} = r_{lj} - s_{lj}$ denoting the change of the number of molecular species $X_{j}$ by the *l*th reaction.

Under well-mixed and dilute conditions, if $P({\mathbf{x}},t)$ denotes the probability for the state vector ${\mathbf{x}} =( {x_{1} ,x_{2} ,\ldots,x_{N} } )$ at time $t$, then the probability evolution of the system (1) can be described by chemical master equation

2 $$\frac{{dP({\mathbf{x}},t)}}{dt} = \sum\limits_{l = 1}^{L} {P({\mathbf{x}} - {\mathbf{S}}_{l} ,t)} a_{l} ({\mathbf{x}} - {\mathbf{S}}_{l} ) - \sum\limits_{l = 1}^{L} {P({\mathbf{x}},t)} a_{l} ({\mathbf{x}})$$

where ${\mathbf{S}}_{l} = ( {S_{l1} , \ldots ,S_{lN} } )$ is the row vector of the stoichiometric matrix and $a_{l} ({\mathbf{x}})$ is the propensity function for the *l*th reaction determined by mass action kinetics. For example, in chemical kinetics^38^, for the reaction $s_{1} X_{1} + s_{2} X_{2} \to r_{1} X_{3} + r_{2} X_{4}$, the reaction rate law expression is

3 $$a(x_{1} ,x_{2} ) = K \cdot x_{1}^{{s_{1} }} \cdot x_{2}^{{s_{2} }}$$

where *K* is the reaction constant.

Then, we establish the stochastic mathematical modeling of making oligomers. Denote the number of $M_{0} ,M_{1} ,M_{2} ,M_{3} ,M_{4} ,M_{5} ,M_{{6}}$ by ${\text{x}}(t) = x_{1} (t),x_{2} (t),x_{3} (t),x_{4} (t),x_{5} (t),x_{{6}} (t),x_{{7}} (t)$ at time $t$. In modeling, the source term of monomers written as $x_{{1}} (t) \to x_{{1}} (t) + {1}$ is also considered and the production rate is assumed to be $k_{p}$. For the system described in Fig.  1, the stoichiometric matrix is

$$S = \left[ {\begin{array}{*{20}c} 1 & \quad { - 1} & \quad 0 & \quad 0 & \quad 0 & \quad 0 & \quad 0 & \quad 1 & \quad 0 & \quad 0 & \quad 0 & \quad 0 & \quad 0 \\ 0 & \quad 1 & \quad { - 2} & \quad { - 1} & \quad { - 1} & \quad { - 1} & \quad { - 1} & \quad { - 1} & \quad 2 & \quad 1 & \quad 1 & \quad 1 & \quad 1 \\ 0 & \quad 0 & \quad 1 & \quad { - 1} & \quad 0 & \quad 0 & \quad 0 & \quad 0 & \quad { - 1} & \quad 1 & \quad 0 & \quad 0 & \quad 0 \\ 0 & \quad 0 & \quad 0 & \quad 1 & \quad { - 1} & \quad 0 & \quad 0 & \quad 0 & \quad 0 & \quad { -1} & \quad { 1} & \quad 0 & \quad 0 \\ 0 & \quad 0 & \quad 0 & \quad 0 & \quad 1 & \quad { - 1} & \quad 0 & \quad 0 & \quad 0 & \quad 0 & \quad -1 & \quad { 1} & \quad 0 \\ 0 & \quad 0 & \quad 0 & \quad 0 & \quad 0 & \quad 1 & \quad { - 1} & \quad 0 & \quad 0 & \quad 0 & \quad 0 & \quad -1 & \quad { 1} \\ 0 & \quad 0 & \quad 0 & \quad 0 & \quad 0 & \quad 0 & \quad 1 & \quad 0 & \quad 0 & \quad 0 & \quad 0 & \quad 0 & \quad -1 \\ \end{array} } \right]^{{\text{T}}}$$,then the chemical master equation of the model is

4 $$\begin{gathered} \frac{{dP(x_{1} ,x_{2} ,x_{3} ,x_{4} ,x_{5} ,x_{6} ,x_{7} ,t)}}{dt} = k_{p} P(x_{1} - 1,x_{2} ,x_{3} ,x_{4} ,x_{5} ,x_{6} ,x_{7} ,t) \hfill \\ \quad + K_{0} (x_{1} + 1)P(x_{1} + 1,x_{2} - 1,x_{3} ,x_{4} ,x_{5} ,x_{6} ,x_{7} ,t) + K_{1} (x_{2} + 2)^{2} P(x_{1} ,x_{2} + 2,x_{3} - 1,x_{4} ,x_{5} ,x_{6} ,x_{7} ,t) \hfill \\ \quad + K_{2} (x_{3} + 1)(x_{2} + 1)P(x_{1} ,x_{2} + 1,x_{3} + 1,x_{4} - 1,x_{5} ,x_{6} ,x_{7} ,t) + K_{3} (x_{4} + 1)(x_{2} + 1) \hfill \\ \quad \times P(x_{1} ,x_{2} + 1,x_{3} ,x_{4} + 1,x_{5} - 1,x_{6} ,x_{7} ,t) + K_{4} (x_{5} + 1)(x_{2} + 1)P(x_{1} ,x_{2} + 1,x_{3} ,x_{4} ,x_{5} + 1,x_{6} - 1,x_{7} ,t) \hfill \\ \quad + K_{5} (x_{6} + 1)(x_{2} + 1)P(x_{1} ,x_{2} + 1,x_{3} ,x_{4} ,x_{5} ,x_{6} + 1,x_{7} - 1,t) \hfill \\ \quad + K_{6} (x_{2} + 1)P(x_{1} - 1,x_{2} + 1,x_{3} ,x_{4} ,x_{5} ,x_{6} ,x_{7} ,t) + K_{{7}} (x_{3} + 1)P(x_{1} ,x_{2} - 2,x_{3} + 1,x_{4} ,x_{5} ,x_{6} ,x_{7} ,t) \hfill \\ \quad + K_{{8}} (x_{4} + 1)P(x_{1} ,x_{2} - 1,x_{3} - 1,x_{4} + 1,x_{5} ,x_{6} ,x_{7} ,t) + K_{{9}} (x_{5} + 1)P(x_{1} ,x_{2} - 1,x_{3} ,x_{4} - 1,x_{5} + 1,x_{6} ,x_{7} ,t) \hfill \\ \quad + K_{{{10}}} (x_{6} + 1)P(x_{1} ,x_{2} - 1,x_{3} ,x_{4} ,x_{5} - 1,x_{6} + 1,x_{7} ,t) + K_{{{11}}} (x_{7} + 1)P(x_{1} ,x_{2} - 1,x_{3} ,x_{4} ,x_{5} ,x_{6} - 1,x_{7} + 1,t) \hfill \\ \quad - (k_{p} + K_{0} x_{1} + K_{1} x_{2} x_{2} + K_{2} x_{2} x_{3} + K_{3} x_{3} x_{4} + K_{4} x_{2} x_{5} + K_{5} x_{2} x_{6} + K_{6} x_{2} + K_{{7}} x_{3} + K_{{8}} x_{{4}} + K_{{9}} x_{{5}} + K_{{{10}}} x_{{6}} + K_{{{11}}} x_{{7}} ) \hfill \\ \quad \times P(x_{1} ,x_{2} ,x_{3} ,x_{4} ,x_{5} ,x_{6} ,x_{7} ,t) \hfill \\ \end{gathered}$$

The above chemical master equation model describes the initial formation of oligomers in a stochastic sense. We remark that it is essential stochastic generalization of a time discrete deterministic model^32^ and a continuous time deterministic counterpart^39^ for the aggregation process. Actually, elegant analytical solutions have been developed to the time continuous model without an upper limit to oligomer size^39^, and the time discrete model can be regarded as its simplification with an upper limit to the oligomer size.

### Moment closure method

Although the chemical master Eq. (4) is a powerful tool for describing the stochastic dynamics of the system (1), it is difficult to find an exact analytic solution. Instead, some simulation techniques such as Gillespie’s stochastic simulation algorithm (SSA) have been presented^40^. But the SSA is relatively expensive in computational cost especially when the system size is large. To overcome this shortcoming, moment closure techniques for approximating the low-order moments have become more and more popular^33,34,41–43^. This is acceptable because the first two order statistical moments (mean and variance or covariance) are sufficient for a decent description of the ensemble dynamics such as averaging behavior and evolution of the noise in the system^41^.

The moment equations corresponding to Eq. (4) is not self-closed since the evolution of the M*th* order moments depend on the (M + 1)*th* order moments. That is, the evolution of the resultant first two order moments involves the third order moments. The so called moment closure is to approximate the involving higher order moments as nonlinear functions of lower-order moments. The frequently adopted closure schemes include Gaussian moment closure, lognormal moment closure, gamma moment closure and binominal moment closure. Here, we apply a kind of lognormal moment closure to Eq. (4) for two reasons. The first reason is that the important features of asymmetry and nonnegativity of the molecular reaction system make that population of species in bio-statistics tends to be lognormal distributed^34^. The second reason is that the lognormal closure scheme, also named the derivative matching closure as presented in Refs.^42,43^, does not necessitate assumptions about the priori distribution. In this method, the nonlinear closure functions are given by matching time derivatives of the unclosed exact moment equations with those of the approximate closed moment equations at some initial point.

Given ${\mathbf{m}} = \left( {m_{1} ,m_{2} ,\ldots,m_{{7}} } \right) \in {\rm N}_{ \ge 0}^{{7}}$, we define moment generating function to be

5 $$M({{\varvec{\uptheta}}},t) = \sum\limits_{{\mathbf{x}}} {e^{{\varvec{\uptheta}{\mathbf{x}}}} P({\mathbf{x}},t)} = \sum\limits_{{{\mathbf{m}} = \mathbf{0}}}^{\infty } {\mu_{{\mathbf{m}}} } \frac{{{{\varvec{\uptheta}}}^{{\mathbf{m}}} }}{{{\mathbf{m}}!}}$$

where ${\mathbf{x}}^{{\mathbf{m}}} : = x_{1}^{{m_{1} }} \cdots x_{{7}}^{{m_{{7}} }}$, $\frac{{{{\varvec{\uptheta}}}^{{\mathbf{m}}} }}{{{\mathbf{m}}!}} = \frac{{\theta_{1}^{{m_{1} }} }}{{m_{1} !}} \times \frac{{\theta_{2}^{{m_{2} }} }}{{m_{2} !}} \times \cdots \times \frac{{\theta_{7}^{{m_{7} }} }}{{m_{7} !}}$, $\mu_{{\mathbf{m}}} = E[{\mathbf{x}}^{{\mathbf{m}}} ] = \sum\limits_{{\mathbf{x}}} {{\mathbf{x}}^{{\mathbf{m}}} P({\mathbf{x}},t)}$ is the raw moment of ${\mathbf{x}}$ associated with ${\mathbf{m}}$ and the sum $\sum\limits_{i = 1}^{{7}} {m_{i} }$ is called the order of the raw moment. Then, multiplying Eq. (4) by $e^{{\varvec{\uptheta}{\mathbf{ x}}}}$ and summing over all possible values of ${\mathbf{x}}$, we obtain

6 $$\frac{d}{dt}\sum\limits_{{\mathbf{x}}} {P({\mathbf{x}},t)e^{{\varvec{\uptheta}{\mathbf{x }}}} } = \sum\limits_{l = 1}^{13} {\left[ {(e^{{\varvec{\uptheta}{\mathbf{ S}}_{l} }} - 1)\sum\limits_{{\mathbf{x}}} {e^{{\varvec{\uptheta}{\mathbf{x }}}} P({\mathbf{x}},t)a_{l} ({\mathbf{x}})} } \right]}$$

The detailed derivation can be found in supplementary method. With the moment generating function (5) in mind, the general form of moment equations is given by rewritten the Eq. (6) and extraction of the coefficients of $\theta_{1} ,\theta_{2} , \ldots ,\theta_{7}$

7 $$\frac{\partial }{\partial t}\mu_{{\mathbf{m}}} = \sum\limits_{l = 1}^{13} {\left[ {\sum\limits_{{\mathbf{i}}} {a_{{l,{\mathbf{i}}}} \sum\limits_{{{\mathbf{k}} = {\mathbf{0}}}}^{{\mathbf{m}}} {{\mathbf{S}}_{l}^{{\mathbf{k}}} \left( {\begin{array}{*{20}c} {\mathbf{m}} \\ {\mathbf{k}} \\ \end{array} } \right)\mu_{{{\mathbf{i}} + {\mathbf{m}}{ - }{\mathbf{k}}}} } } } \right]} - \sum\limits_{{\mathbf{i}}} {\sum\limits_{l = 1}^{13} {a_{{l,{\mathbf{i}}}} } \mu_{{{\mathbf{i}} + {\mathbf{m}}}} }$$

where $a_{{l,{\mathbf{i}}}}$ is the multinomial coefficient of $a_{l} ({\mathbf{x}})$.

Since we mainly focus on the important stochastic quantities namely mean and variance, the system of $2C_{7}^{1} + C_{7}^{2} = 35$ equations for the first two order moments are derived shown in supplementary equations, from which we can see these equations depending on third order moments. For calculating, we truncate the infinite hierarchy (7) to a finite-dimensional system by the lognormal closure scheme. Following the lognormal closure scheme^43^, the third order moments can be expressed by the nonlinear function of first two order moments. Assume $\mu_{{{\overline{\mathbf{m}}}}}$(${\overline{\mathbf{m}}} = (\overline{m}_{1} , \ldots ,\overline{m}_{7} )$) be one of the third order moments, then we can find a suitable closure function $\phi_{{({\overline{\mathbf{m}}})}} ( \cdot )$ with the separable form

8 $$\mu_{{{\overline{\mathbf{m}}}}} \approx \phi_{{({\overline{\mathbf{m}}})}} ({{\varvec{\upmu}}}) = \prod\limits_{p = 1}^{s} {(\mu_{{{\mathbf{m}}_{p} }} )^{{\gamma_{p} }} }$$

where ${{\varvec{\upmu}}} = [\mu_{{{\mathbf{m}}_{1} }} , \ldots ,\mu_{{{\mathbf{m}}_{s} }} ]$ is the vector of the first two order moments and $\gamma_{1} , \ldots ,\gamma_{s}$ are chosen as the unique solution of the following set of linear equations

9 $$\left( {\begin{array}{*{20}c} {{\overline{\mathbf{m}}}} \\ {{\mathbf{m}}_{q} } \\ \end{array} } \right) = \sum\limits_{p = 1}^{s} {\gamma_{p} } \left( {\begin{array}{*{20}c} {{\mathbf{m}}_{p} } \\ {{\mathbf{m}}_{q} } \\ \end{array} } \right) \quad q = 1, \ldots ,s$$

With the closure functions in Eq. (8) available, all the involving third-order moments can be expressed by the first two order moments, thus a self-closed moment system consisting of thirty-five ordinary differential equations can be deduced from Eq. (7). The detailed deduction based on the lognormal closure scheme (8) can be found from the supplementary. We emphasize that all the deductions are implemented by hand. We then apply the fourth order Ronge-Kutta method to the closed moment system and the first-order and the second-order moments are solved.

## Results

It is generally hard to determine the unknown rate constants of oligomer growth. For simplicity, we assume all the polymerization rates are identical, namely $K_{1} = K_{2} = K_{3} = K_{4} = K_{5} = K_{a}$ and all the polymerization rates are the same, i.e. $K_{7} = K_{8} = K_{9} = K_{10} = K_{11} = K_{b}$. Although the identical assumption as an approximation to the nonlinear system is not that realistic, our numerical experience shows that the accuracy of the lognormal closure scheme does not rely on the relative variation of these rate parameters. As oligomeric intermediates in fibril formation are thermodynamically unstable^24,44,45^, we specify equilibrium constant $k_{e} = {{K_{a} } / {K_{b} }}$ around 0.02, which ensures oligomers are suitably unstable compared to monomers. Since monomers are usually not produced in vitro experiments, we set the rate of monomer production $k_{p} = 0$. We assume the refolding rate ($K_{6}$) is larger than misfolding rate ($K_{0}$) in all the simulation. The initial conditions for the seven species are taken as $x_{2} (0) = \ldots = x_{{7}} (0) = 1$ and $x_{1} (0) = 2000$ not including Fig. 3.

**Figure 2:**
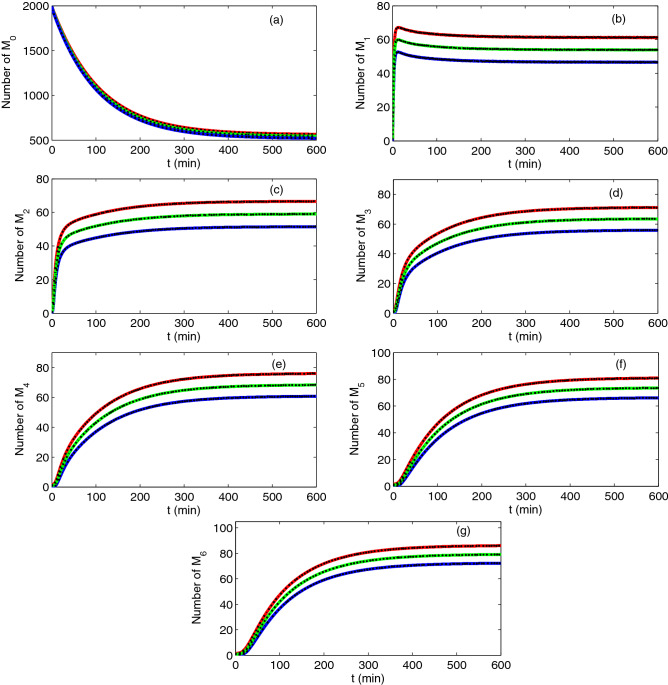
The time evolution of the number of $M_{{0}} ,M_{1} ,M_{2} ,\ldots,M_{{6}}$. Comparison of the mean + standard deviation (upper red curve), mean (middle green curve) and mean − standard deviation (lower blue curve) by moment closure method (solid curves) and SSA (block dots). The initial condition is $x_{1} (0) = 2000$, $x_{2} (0) = \ldots = x_{{7}} (0) = 1$ and parameter values are $k_{p} = 0$, $K_{0} = 0.01\min^{ - 1}$, $K_{a} = 0.002\min^{ - 1}$, $K_{6} = 0.1\min^{ - 1}$, $K_{b} = 0.1\min^{ - 1}$. The results by SSA are based on 10,000 realizations.

Figure 2 shows the mean and the standard deviation we obtain for monomers, misfolded monomers, diamers, … and hexamers. It can be seen that the results derived from the lognormal closure are in good agreement with the results obtained from the SSA realization. This coincidence implies that the moment method is efficient and accurate in capturing the time evolution of mean and standard deviation. Thus, we only show the results obtained by the moment closure method in the other figures. As seen from the figure, all the reaction species can evolve into a steady state for suitable parameters.

**Figure 3:**
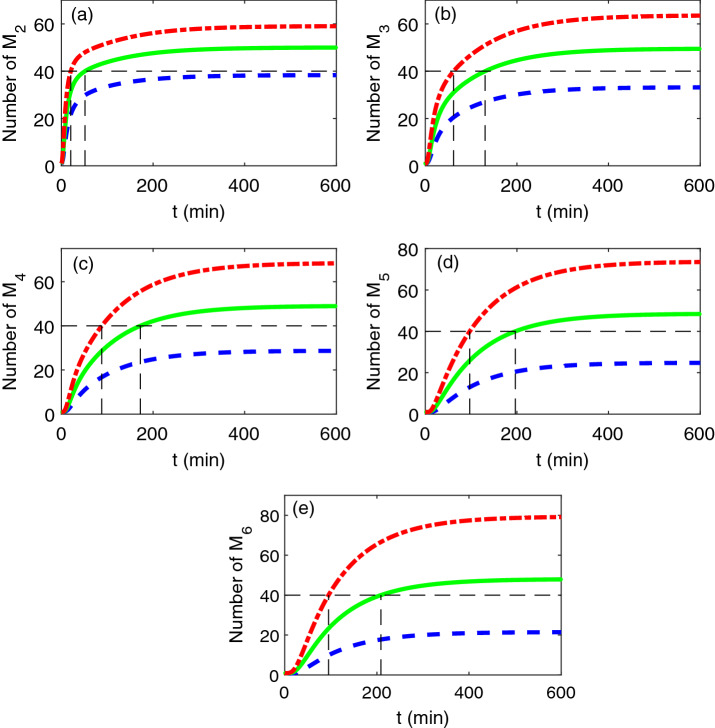
The time evolution of the number of different tapes of oligomers obtained by the moment closure method for different initial number of monomers $x_{1} (0)$:$x_{1} (0) = 1000$ (blue broken line), $x_{1} (0) = 1500$ (green solid line) and $x_{1} (0) = 2000$ (red dotted dash line). The horizontal dotted line represents a given level of the number of oligomers, while the vertical dotted line represents the time required to reach the given level. The other parameters are same as Fig. 2. The results by SSA are based on 10,000 realizations.

Figure 3 shows that the quantitative evolution of the numbers of diamers, …, hexamers under different initial numbers of the monomers. It is shown from the picture that the initial number of monomers has same effect on different tapes of oligomers. That is, as the initial number of the monomers increases, the number of oligomers increases and the difference among initial values also undergoes a transient increase. This result implies that increasing the initial number of the monomers will speed up the process of nucleation phase. This observation supports the statement in Ref.^21^ that the aggregation process is highly dependent on the number of monomers. Note that the number of the monomers of transthyretin is typically at steady state but may vary from person to person, thus the observation from Fig. 3 may be helpful in explaining why some people are more apt to suffer from transthyretin amyloid disease under same condition to some extent.

**Figure 4:**
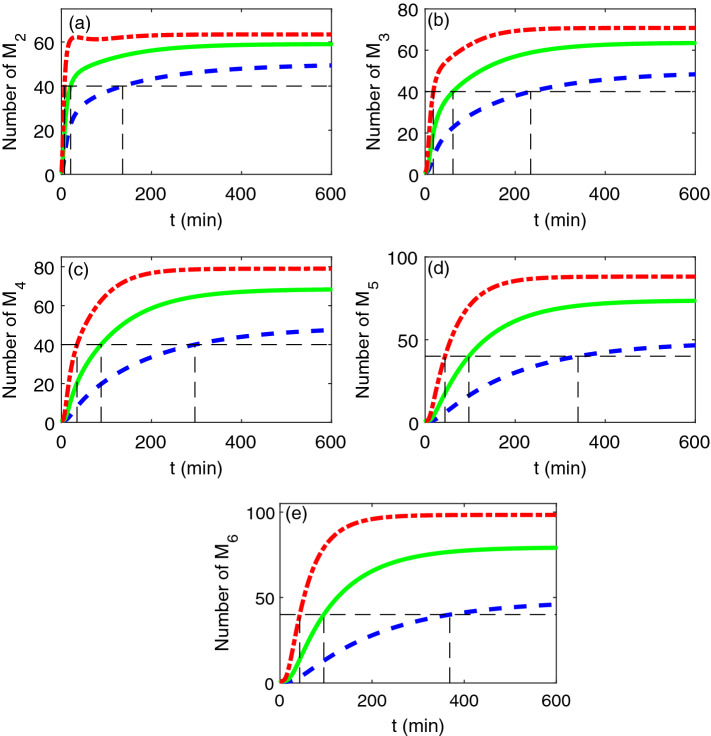
The time evolution of the number of different tapes of oligomers obtained by the moment closure method for different misfolding rate $K_{{0}}$:$K_{{0}} = 0.005$ (blue broken line), $K_{{0}} = 0.01$ (green solid line) and $K_{{0}} = 0.02$ (red dotted dash line). The horizontal dotted line represents a given level of the number of oligomers, while the vertical dotted line represents the time required to reach the given level. The other parameters are same as Fig. 2. The results by SSA are based on 10,000 realizations.

Figure 4 shows the influence of misfolding rate on the number of oligomers. This qualitative observation in Fig. 4 demonstrates that the misfolding rate has a major impact on the aggregation process, that is, as the misfolding rate increases, all the five types of oligomers grow in number, and the time for these oligomers to reach a given level also becomes shortened. Since the toxic oligomers accumulation plays a central role in amyloidoses^46^, our observation suggests that decreasing the misfolding rate should be useful in slowing or inhibiting the formation of transthyretin amyloid disease. In fact, there have been some literatures towards to reduce the misfloding rate of proteins^47,48^.

## Conclusion

It is well known that amyloidal aggregation is the hallmark of amyloidoses. In order to quantitatively explore the process of early amyloidal aggregation, we have developed a stochastic mathematical model about oligomers aggregation from monomers using rate law in chemical kinetics. We have adopted a typical moment method based on lognormal closure to capture the statistical moments, and massive calculations show very good agreement between the semi-analytic method and the Gillespie’s SSA for the stochastic model. Our results show that the aggregation of monomers into oligomers is highly dependent on the number of monomers and the misfolding rate, and decreasing the number of monomers and the misfolding rate can inhibit the formation of the toxic oligomers. Our research may be helpful in explaining the individual variation in suffering from transthyretin amyloid disease and emphasizes the importance of controlling the misfolding of protein in preventing this type of disease.

Additionally, we remark that the lognormal moment method is successful for stochastic master equation relevant to the transthyretin with an upper limit of oligomer size $N = 6$, and we expect this method would also be effective with a different upper limit which might be relevant to other amyloidogenic systems. The method in the present work can provide essential quantitative insights into the mechanism of the early steps in the aggregation reactions. The mechanism behind the formation of amyloidoses is far from clear, and in the future we will continue to investigate the kinetic of amyloid nucleation and the mechanism of amyloid fibril growth.

## Supplementary information

Supplementary Information
