# Supplementary material for: Stochastic master equation for early protein aggregation in the transthyretin amyloid disease
Source: Sci Rep. 2020 Jul 24;10:12437. doi: 10.1038/s41598-020-69319-x (PMC7381670; doi:10.1038/s41598-020-69319-x)
Supplement: Supplementary file 1 — Supplementary Information [file 41598_2020_69319_MOESM1_ESM.docx]

**Stochastic master equation for early protein aggregation** **in the transthyretin amyloid disease**

Ruo-Nan Liu and Yan-Mei Kang^*^

School of Mathematics and Statistics, Xi’an Jiaotong University, Xi’an, Shaanxi, 710049, China

^*^Corresponding author email: ymkang@xjtu.edu.cn

**Supplementary Method**

**1. The detailed derivation of Eq. (6)**

In deriving the Eq. (6), we first rewrite the Eq. (4) as

$\frac{dP(x,t)}{dt}=\sum_{l=1}^{13} P(x-S_{l},t)a_{l}(x-S_{l})-\sum_{l=1}^{13} P(x,t)a_{l}(x)$,

Then multiplying Eq. (4) by$e^{\theta x}$and summing over all possible values of$x$, we have

$\frac{d}{dt}\sum_{x} P(x,t)e^{x\theta}=\sum_{x} e^{x\theta}\sum_{l=1}^{13} P(x-S_{l},t)a_{l}(x-S_{l})-\sum_{x} e^{x\theta}\sum_{l=1}^{13} P(x,t)a_{l}(x)$. (S1)

The first term in right hand side can be converted to

$$\sum_{x} e^{x\theta}\sum_{l=1}^{13} P(x-S_{l},t)a_{l}(x-S_{l})=\sum_{l=1}^{13} \sum_{x} e^{(x+S_{l})\theta}P(x,t)a_{l}(x)$$

Substitution the above equation to Eq.(S1), we can obtain

$\frac{d}{dt}\sum_{x} P(x,t)e^{x\theta}=\sum_{l=1}^{13} \left[(e^{\theta S_{l}}-1)\sum_{x} e^{x\theta}P(x,t)a_{l}(x)\right]$.

**2. Lognormal closure scheme**

Assume $\mu_{\bar{m}}$() be one of the third order moments, then we can find a suitable closure function $\phi_{(\bar{m}}(\cdot)$such that with $\mu\text{=[}\mu_{m_{1}},\ldots,\mu_{m_{s}}]$ being a row vector of the first two order moments. According to the lognormal closure scheme [1], the closure function $\phi_{(\bar{m}}(\cdot)$ is constructed with the separable form

$\phi_{(\bar{m}}(\mu)=\prod_{p=1}^{s} (\mu_{m_{p}})^{\gamma_{p}}$, (S2)

by matching derivatives of the original unclosed moment equations with those of the approximate closed moment equations for some initial time and set of initial conditions. Namely, for every initial condition $x(t_{0})=x_{0}$with probability one, constants$\gamma_{1},\ldots\gamma_{s}$are chosen by

(S3)

(S4)

where$\varepsilon(x_{0})$is polynomial in $x_{0}$ of degree 2 which is dominated over by $\left.\frac{d^{2}\mu_{m_{i}}(t)}{dt^{2}}\right|_{t=t_{0}}$with $\mu_{m_{i}}(t)$ representing a moments of order *M*.

For the initial condition $x(t_{0})=x_{0}$with probability one, by the requirement (S3), there holds

$x_{0}^{\bar{m}}=\prod_{p=1}^{s} (x_{0}^{m_{p}})^{\gamma_{p}}=x_{0}^{\sum_{p=1}^{s} \gamma_{p}m_{p}}$.

Thus, for$\sum_{j} m_{q_{j}}=1$, we have

. (S5)

In order to fulfill the requirement (S4), we can rewrite Eq. (7) for the first two order moments as

$\left.\frac{d\mu_{m}(t)}{dt}\right|_{t=t_{0}}=\sum_{l=1}^{13} \sum_{i} a_{l,i}\left[\sum_{q=1}^{s} S_{l}^{m_{q}}\left(\begin{matrix} m \\ m_{q} \end{matrix}\right)x_{0}^{i+m\text{-}m_{q}}\right]$ (S6)

with$m=(m_{1},\ldots,m_{7})$. And for with

(S7)

where$\alpha_{l}$is constant.

By means of Eq. (S2) and Eq. (S6),

(S8)

Then, according to Eq. (S4), comparing Eq. (S7) and (S8), we can choose $\gamma_{1},\ldots\gamma_{s}$ as the unique solution of the following set of linear equations

, $q=1,\ldots,s$, (S9)

with$\varepsilon(x_{0})=\sum_{l=1}^{L} a_{l}(x_{0})\alpha_{l}$.

The lognormal closure scheme can be demonstrated by a concrete example. We assume that the third order statistical moment $E(x^{3})$ can be expressed by the first two order moments in the separable form $E(x^{3})\cong E(x)^{\gamma_{1}}E(x^{2})^{\gamma_{2}}$ where$\gamma_{i} (i=1, 2)$ satisfy$\left(\begin{matrix} 3 \\ 1 \end{matrix}\right)=\left(\begin{matrix} 1 \\ 1 \end{matrix}\right)\gamma_{1}+\left(\begin{matrix} 2 \\ 1 \end{matrix}\right)\gamma_{2}$, $\left(\begin{matrix} 3 \\ 2 \end{matrix}\right)=\left(\begin{matrix} 1 \\ 2 \end{matrix}\right)\gamma_{1}+\left(\begin{matrix} 2 \\ 2 \end{matrix}\right)\gamma_{2}$. Namely, $E(x^{3})\cong\frac{E(x^{2})^{3}}{E(x)^{3}}$.

For detail, the moment closure functions of two other third order moments can be expressed in terms of the ﬁrst two order moments as follows:

$E(x_{1}^{2}x_{2})\cong\frac{E(x_{1}^{2})E(x_{1}x_{2})^{2}}{E(x_{2})E(x_{1})^{2}}$, (S10)

$E(x_{1}x_{2}x_{3})\cong\frac{E(x_{1}x_{2})E(x_{1}x_{3})E(x_{2}x_{3})}{E(x_{1})E(x_{2})E(x_{3})}$. (S11)

**Supplementary Equations**

The original moment system of the first two order moments is a system of $2C_{7}^{1}+C_{7}^{2}$equations, given by

$$\frac{\partial}{\partial t}\mu_{1000000}=k_{p}-K_{0}\mu_{1000000}+K_{6}\mu_{0100000}$$

$$\begin{matrix} \frac{\partial}{\partial t}\mu_{0100000}=K_{0}\mu_{1000000}-2K_{1}\mu_{0200000}-K_{2}\mu_{0110000}-K_{3}\mu_{0101000}-K_{4}\mu_{0100100}-K_{5}\mu_{0100010} \\ -K_{6}\mu_{0100000}+2K_{7}\mu_{0010000}+K_{8}\mu_{0001000}+K_{9}\mu_{0000100}+K_{10}\mu_{0000010}+K_{11}\mu_{0000001} \end{matrix}$$

$$\frac{\partial}{\partial t}\mu_{0010000}=K_{1}\mu_{0200000}-K_{2}\mu_{0110000}-K_{7}\mu_{0010000}+K_{8}\mu_{0001000}$$

$$\frac{\partial}{\partial t}\mu_{0001000}=K_{2}\mu_{0110000}-K_{3}\mu_{0101000}-K_{8}\mu_{0001000}+K_{9}\mu_{0000100}$$

$$\frac{\partial}{\partial t}\mu_{0000100}=K_{3}\mu_{0101000}-K_{4}\mu_{0100100}-K_{9}\mu_{0000100}+K_{10}\mu_{0000010}$$

$$\frac{\partial}{\partial t}\mu_{0000010}=K_{4}\mu_{0100100}-K_{5}\mu_{0100010}-K_{10}\mu_{0000010}+K_{11}\mu_{0000001}$$

$$\frac{\partial}{\partial t}\mu_{0000001}=K_{5}\mu_{0100010}-K_{11}\mu_{0000001}$$

$$\frac{\partial}{\partial t}\mu_{2000000}=k_{p}+2k_{p}\mu_{1000000}-2K_{0}\mu_{2000000}+K_{0}\mu_{1000000}+2K_{6}\mu_{1100000}+K_{6}\mu_{0100000}$$

$$\begin{matrix} \frac{\partial}{\partial t}\mu_{0200000}=2K_{0}\mu_{1100000}+K_{0}\mu_{1000000}-4K_{1}\mu_{0300000}+4K_{1}\mu_{0200000}-2K_{2}\mu_{0210000}+K_{2}\mu_{0110000} \\ -2K_{3}\mu_{0201000}+K_{3}\mu_{0101000}-2K_{4}\mu_{0200100}+K_{4}\mu_{0100100}-2K_{5}\mu_{0200010} \\ +K_{5}\mu_{0100010}-2K_{6}\mu_{0200000}+K_{6}\mu_{0100000}+4K_{7}\mu_{0110000}+4K_{7}\mu_{0010000} \\ +2K_{8}\mu_{0101000}+K_{8}\mu_{0001000}+2K_{9}\mu_{0100100}+K_{9}\mu_{0000100}+2K_{10}\mu_{0100010} \\ +K_{10}\mu_{0000010}+2K_{11}\mu_{0100001}+K_{11}\mu_{0000001} \end{matrix}$$

$$\begin{matrix} \frac{\partial}{\partial t}\mu_{0020000}=2K_{1}\mu_{0210000}+K_{1}\mu_{0200000}-2K_{2}\mu_{0120000}+K_{2}\mu_{0110000}-2K_{7}\mu_{0020000}+K_{7}\mu_{0010000} \\ +2K_{8}\mu_{0011000}+K_{8}\mu_{0001000} \end{matrix}$$

$$\begin{matrix} \frac{\partial}{\partial t}\mu_{0002000}=2K_{2}\mu_{0111000}+K_{2}\mu_{0110000}-2K_{3}\mu_{0102000}+K_{3}\mu_{0101000}-2K_{8}\mu_{0002000}+K_{8}\mu_{0001000} \\ +2K_{9}\mu_{0001100}+K_{9}\mu_{0000100} \end{matrix}$$

$$\begin{matrix} \frac{\partial}{\partial t}\mu_{0000200}=2K_{3}\mu_{0101100}+K_{3}\mu_{0101000}-2K_{4}\mu_{0100200}+K_{4}\mu_{0100100}-2K_{9}\mu_{0000200}+K_{9}\mu_{0000100} \\ +2K_{10}\mu_{0000110}+K_{10}\mu_{0000010} \end{matrix}$$

$$\begin{matrix} \frac{\partial}{\partial t}\mu_{0000020}=2K_{4}\mu_{0100110}+K_{4}\mu_{0100100}-2K_{5}\mu_{0100020}+K_{5}\mu_{0100010}-2K_{10}\mu_{0000020}+K_{10}\mu_{0000010} \\ +2K_{11}\mu_{0000011}+K_{11}\mu_{0000001} \end{matrix}$$

$$\frac{\partial}{\partial t}\mu_{0000002}=2K_{5}\mu_{0100011}+K_{5}\mu_{0100010}-2K_{11}\mu_{0000002}+K_{11}\mu_{0000001}$$

$$\begin{matrix} \frac{\partial}{\partial t}\mu_{1100000}=k_{p}\mu_{0100000}-K_{0}\mu_{1100000}+K_{0}\mu_{2000000}-K_{0}\mu_{1000000}-2K_{1}\mu_{1200000}-K_{2}\mu_{1110000} \\ -K_{3}\mu_{1101000}-K_{4}\mu_{1100100}-K_{5}\mu_{1100010}+K_{6}\mu_{0200000}-K_{6}\mu_{1100000}-K_{6}\mu_{0100000} \\ +2K_{7}\mu_{1010000}+K_{8}\mu_{1001000}+K_{9}\mu_{1000100}+K_{10}\mu_{1000010}+K_{11}\mu_{1000001} \end{matrix}$$

$$\begin{matrix} \frac{\partial}{\partial t}\mu_{1010000}=k_{p}\mu_{0010000}-K_{0}\mu_{1010000}+K_{1}\mu_{1200000}-K_{2}\mu_{1110000}+K_{6}\mu_{0110000}-K_{7}\mu_{1010000} \\ +K_{8}\mu_{1001000} \end{matrix}$$

$$\begin{matrix} \frac{\partial}{\partial t}\mu_{1001000}=k_{p}\mu_{0001000}-K_{0}\mu_{1001000}+K_{2}\mu_{1110000}-K_{3}\mu_{1101000}+K_{6}\mu_{0101000}-K_{8}\mu_{1001000} \\ +K_{9}\mu_{1000100} \end{matrix}$$

$$\begin{matrix} \frac{\partial}{\partial t}\mu_{1000100}=k_{p}\mu_{0000100}-K_{0}\mu_{1000100}+K_{3}\mu_{1101000}-K_{4}\mu_{1100100}+K_{6}\mu_{0100100}-K_{9}\mu_{1000100} \\ +K_{10}\mu_{1000010} \end{matrix}$$

$$\begin{matrix} \frac{\partial}{\partial t}\mu_{1000010}=k_{p}\mu_{0000010}-K_{0}\mu_{1000010}+K_{4}\mu_{1100100}-K_{5}\mu_{1100010}+K_{6}\mu_{0100010}-K_{10}\mu_{1000010} \\ +K_{11}\mu_{1000001} \end{matrix}$$

$$\frac{\partial}{\partial t}\mu_{1000001}=k_{p}\mu_{0000001}-K_{0}\mu_{1000001}+K_{5}\mu_{1100010}+K_{10}\mu_{0100001}-K_{11}\mu_{1000001}$$

$$\begin{matrix} \frac{\partial}{\partial t}\mu_{0110000}=K_{0}\mu_{1010000}-2K_{1}\mu_{0210000}+K_{1}\mu_{0300000}-2K_{1}\mu_{0200000}-K_{2}\mu_{0120000}-K_{2}\mu_{0210000} \\ +K_{2}\mu_{0110000}-K_{3}\mu_{0111000}-K_{4}\mu_{0110100}-K_{5}\mu_{0110010}-K_{6}\mu_{0110000}+2K_{7}\mu_{0020000} \\ -K_{7}\mu_{0110000}-2K_{7}\mu_{0010000}+K_{8}\mu_{0011000}+K_{8}\mu_{0101000}+K_{8}\mu_{0001000}+K_{9}\mu_{0010100} \\ +K_{10}\mu_{0010010}+K_{11}\mu_{0010001} \end{matrix}$$

$$\begin{matrix} \frac{\partial}{\partial t}\mu_{0101000}=K_{0}\mu_{1001000}-2K_{1}\mu_{0201000}-K_{2}\mu_{0111000}+K_{2}\mu_{0210000}-K_{2}\mu_{0110000}-K_{3}\mu_{0102000} \\ -K_{3}\mu_{0201000}+K_{3}\mu_{0101000}-K_{4}\mu_{0101100}-K_{5}\mu_{0101010}-K_{6}\mu_{0101000}+2K_{7}\mu_{0011000} \\ +K_{8}\mu_{0002000}-K_{8}\mu_{0101000}-K_{8}\mu_{0001000}+K_{9}\mu_{0001100}+K_{9}\mu_{0100100}+K_{9}\mu_{0000100} \\ +K_{10}\mu_{0001010}+K_{11}\mu_{0001001} \end{matrix}$$

$$\begin{matrix} \frac{\partial}{\partial t}\mu_{0100100}=K_{0}\mu_{1000100}-2K_{1}\mu_{0200100}-K_{2}\mu_{0110100}-K_{3}\mu_{0101100}+K_{3}\mu_{0201000}-K_{3}\mu_{0101000} \\ -K_{4}\mu_{0100200}-K_{4}\mu_{0200100}+K_{4}\mu_{0100100}-K_{5}\mu_{0100110}-K_{6}\mu_{0100100}+2K_{7}\mu_{0010100} \\ +K_{8}\mu_{0001100}+K_{9}\mu_{0000200}-K_{9}\mu_{0100100}-K_{9}\mu_{0000100}+K_{10}\mu_{0000110}+K_{10}\mu_{0100010} \\ +K_{10}\mu_{0000010}+K_{11}\mu_{0000101} \end{matrix}$$

$$\begin{matrix} \frac{\partial}{\partial t}\mu_{0100010}=K_{0}\mu_{1000010}-2K_{1}\mu_{0200010}-K_{2}\mu_{0110010}-K_{3}\mu_{0101010}-K_{4}\mu_{0100110}+K_{4}\mu_{0200100} \\ -K_{4}\mu_{0100100}-K_{5}\mu_{0100020}-K_{5}\mu_{0200010}+K_{5}\mu_{0100010}-K_{6}\mu_{0100010}+2K_{7}\mu_{0010010} \\ +K_{8}\mu_{0001010}+K_{9}\mu_{0000110}+K_{10}\mu_{0000020}-K_{10}\mu_{0100010}-K_{10}\mu_{0000010}+K_{11}\mu_{0000011} \\ +K_{11}\mu_{0100001}+K_{11}\mu_{0000001} \end{matrix}$$

$$\begin{matrix} \frac{\partial}{\partial t}\mu_{0100001}=K_{0}\mu_{1000001}-2K_{1}\mu_{0200001}-K_{2}\mu_{0110001}-K_{3}\mu_{0101001}-K_{4}\mu_{0100101}-K_{5}\mu_{0100011} \\ +K_{5}\mu_{0200010}-K_{5}\mu_{0100010}-K_{6}\mu_{0100001}+2K_{7}\mu_{0010001}+K_{8}\mu_{0001001}+K_{9}\mu_{0000101} \\ +K_{10}\mu_{0000011}+K_{11}\mu_{0000002}-K_{11}\mu_{0100001}-K_{11}\mu_{0000001} \end{matrix}$$

$$\begin{matrix} \frac{\partial}{\partial t}\mu_{0011000}=K_{1}\mu_{0201000}-K_{2}\mu_{0111000}+K_{2}\mu_{0120000}-K_{2}\mu_{0110000}-K_{3}\mu_{0111000}-K_{7}\mu_{0011000} \\ +K_{8}\mu_{0002000}-K_{8}\mu_{0011000}-K_{8}\mu_{0001000}+K_{9}\mu_{0010100} \end{matrix}$$

$$\begin{matrix} \frac{\partial}{\partial t}\mu_{0010100}=K_{1}\mu_{0200100}-K_{2}\mu_{0110100}+K_{3}\mu_{0111000}-K_{4}\mu_{0110100}-K_{7}\mu_{0010100}+K_{8}\mu_{0001100} \\ -K_{9}\mu_{0010100}+K_{10}\mu_{0010010} \end{matrix}$$

$$\begin{matrix} \frac{\partial}{\partial t}\mu_{0010010}=K_{1}\mu_{0200010}-K_{2}\mu_{0110010}+K_{4}\mu_{0110100}-K_{5}\mu_{0110010}-K_{7}\mu_{0010010}+K_{8}\mu_{0001010} \\ -K_{10}\mu_{0010010}+K_{11}\mu_{0010001} \end{matrix}$$

$$\frac{\partial}{\partial t}\mu_{0010001}=K_{1}\mu_{0200001}-K_{2}\mu_{0110001}+K_{5}\mu_{0110010}-K_{7}\mu_{0010001}+K_{8}\mu_{0001001}-K_{11}\mu_{0010001}$$

$$\begin{matrix} \frac{\partial}{\partial t}\mu_{0001100}=K_{2}\mu_{0110100}-K_{3}\mu_{0101100}+K_{3}\mu_{0102000}-K_{3}\mu_{0101000}-K_{4}\mu_{0101100}-K_{8}\mu_{0001100} \\ +K_{9}\mu_{0000200}-K_{9}\mu_{0001100}-K_{9}\mu_{0000100}+K_{10}\mu_{0001010} \end{matrix}$$

$$\begin{matrix} \frac{\partial}{\partial t}\mu_{0001010}=K_{2}\mu_{0110010}-K_{3}\mu_{0101010}+K_{4}\mu_{0101100}-K_{5}\mu_{0101010}-K_{8}\mu_{0001010}+K_{9}\mu_{0000110} \\ -K_{10}\mu_{0001010}+K_{11}\mu_{0001001} \end{matrix}$$

$$\frac{\partial}{\partial t}\mu_{0001001}=K_{2}\mu_{0110001}-K_{3}\mu_{0101001}+K_{5}\mu_{0101010}-K_{8}\mu_{0001001}+K_{9}\mu_{0000101}-K_{11}\mu_{0001001}$$

$$\begin{matrix} \frac{\partial}{\partial t}\mu_{0000110}=K_{3}\mu_{0101010}-K_{4}\mu_{0100110}+K_{4}\mu_{0100200}-K_{4}\mu_{0100100}-K_{5}\mu_{0100110}-K_{9}\mu_{0000110} \\ +K_{10}\mu_{0000020}-K_{10}\mu_{0000110}-K_{10}\mu_{0000010}+K_{11}\mu_{0000101} \end{matrix}$$

$$\frac{\partial}{\partial t}\mu_{0000101}=K_{3}\mu_{0101001}-K_{4}\mu_{0100101}+K_{5}\mu_{0100110}-K_{9}\mu_{0000101}+K_{10}\mu_{0000011}-K_{11}\mu_{0000101}$$

$$\begin{matrix} \frac{\partial}{\partial t}\mu_{0000011}=K_{4}\mu_{0100101}-K_{5}\mu_{0100011}+K_{5}\mu_{0100020}-K_{5}\mu_{0100010}-K_{10}\mu_{0000011}-K_{11}\mu_{0000011} \\ +K_{11}\mu_{0000002}-K_{11}\mu_{0000001} \end{matrix}$$

The system of the above 35 equations is not closed since the evolution of the involving third order moments is not contained. According to the lognormal closure scheme [1], the third order moments must be replaced by the following functions of lower-order moments to acquire a self-closed system.

$\mu_{0300000}=\frac{\mu_{0200000}^{3}}{\mu_{0100000}^{3}}$; $\mu_{0210000}=\frac{\mu_{0200000}\mu_{0110000}^{2}}{\mu_{0010000}\mu_{0100000}^{2}}$, $\mu_{0201000}=\frac{\mu_{0200000}\mu_{0101000}^{2}}{\mu_{0001000}\mu_{0100000}^{2}}$,

$\mu_{0200100}=\frac{\mu_{0200000}\mu_{0100100}^{2}}{\mu_{0000100}\mu_{0100000}^{2}}$,$\mu_{0200010}=\frac{\mu_{0200000}\mu_{0100010}^{2}}{\mu_{0000010}\mu_{0100000}^{2}}$,$\mu_{0200001}=\frac{\mu_{0200000}\mu_{0100001}^{2}}{\mu_{0000001}\mu_{0100000}^{2}}$,

$\mu_{1200000}=\frac{\mu_{0200000}\mu_{1100000}^{2}}{\mu_{1000000}\mu_{0100000}^{2}}$,$\mu_{0120000}=\frac{\mu_{0020000}\mu_{0110000}^{2}}{\mu_{0100000}\mu_{0010000}^{2}}$,$\mu_{0102000}=\frac{\mu_{0002000}\mu_{0101000}^{2}}{\mu_{0100000}\mu_{0001000}^{2}}$,

$\mu_{0100200}=\frac{\mu_{0000200}\mu_{0100100}^{2}}{\mu_{0100000}\mu_{0000100}^{2}}$,$\mu_{0100020}=\frac{\mu_{0000020}\mu_{0100010}^{2}}{\mu_{0100000}\mu_{0000010}^{2}}$;$\mu_{0111000}=\frac{\mu_{0110000}\mu_{0011000}\mu_{0101000}}{\mu_{0100000}\mu_{0010000}\mu_{0001000}}$,

$\mu_{0110100}=\frac{\mu_{0110000}\mu_{0010100}\mu_{0100100}}{\mu_{0100000}\mu_{0010000}\mu_{0000100}}$,$\mu_{0110010}=\frac{\mu_{0110000}\mu_{0010010}\mu_{0100010}}{\mu_{0100000}\mu_{0010000}\mu_{0000010}}$,

$\mu_{0110001}=\frac{\mu_{0110000}\mu_{0010001}\mu_{0100001}}{\mu_{0100000}\mu_{0010000}\mu_{0000001}}$,$\mu_{0101100}=\frac{\mu_{0101000}\mu_{0001100}\mu_{0100100}}{\mu_{0100000}\mu_{0001000}\mu_{0000100}}$,

$\mu_{0101010}=\frac{\mu_{0101000}\mu_{0001010}\mu_{0100010}}{\mu_{0100000}\mu_{0001000}\mu_{0000010}}$,$\mu_{0101001}=\frac{\mu_{0101000}\mu_{0001001}\mu_{0100001}}{\mu_{0100000}\mu_{0001000}\mu_{0000001}}$,

$\mu_{0100110}=\frac{\mu_{0100100}\mu_{0000110}\mu_{0100010}}{\mu_{0100000}\mu_{0000100}\mu_{0000010}}$,$\mu_{0100101}=\frac{\mu_{0100100}\mu_{0000101}\mu_{0100001}}{\mu_{0100000}\mu_{0000100}\mu_{0000001}}$,

$\mu_{0100011}=\frac{\mu_{0100010}\mu_{0000011}\mu_{0100001}}{\mu_{0100000}\mu_{0000010}\mu_{0000001}}$.

**Reference**

1. Singh, A. & Hespanha, J. P. Approximate moment dynamics for chemically reacting systems. *IEEE T. Automat. Contr.* **56**(2), 414-418 (2011).
